# Longitudinal evaluation of adherence, retention, and transition patterns of adolescents living with HIV in Nigeria

**DOI:** 10.1371/journal.pone.0236801

**Published:** 2020-07-31

**Authors:** Seema T. Meloni, Patricia Agaba, Charlotte A. Chang, Esther Yiltok, Stephen Oguche, Emeka Ejeliogu, Oche Agbaji, Prosper Okonkwo, Phyllis J. Kanki

**Affiliations:** 1 Department of Immunology and Infectious Diseases, Harvard T.H. Chan School of Public Health, Boston, Massachusetts, United States of America; 2 Department of Family Medicine, University of Jos and Jos University Teaching Hospital, Jos, Nigeria; 3 Department of Pediatrics, University of Jos and Jos University Teaching Hospital, Jos, Nigeria; 4 Department of Medicine, University of Jos and Jos University Teaching Hospital, Jos, Nigeria; 5 APIN Public Health Initiatives, Ltd./Gte., Abuja, Nigeria; Boston University School of Public Health, UNITED STATES

## Abstract

**Introduction:**

Adherence to antiretroviral therapy (ART) and retention in treatment programs are required for successful virologic suppression and treatment outcomes. As the number of adolescents living with HIV continues to increase globally, more information about adherence and retention patterns during and through transition from child- to adult-centered care is needed to ensure provision of a high level of care and inform development of targeted interventions to improve patient outcomes in this vulnerable population. In this analysis, we sought to describe long-term trends in adherence, retention, and virologic suppression in adolescents receiving ART at a pediatric HIV clinic in Nigeria through transition to the adult clinic.

**Setting:**

The Jos University Teaching Hospital, United States President’s Emergency Plan for AIDS Relief (PEPFAR)-funded HIV clinic in Jos, Plateau State, Nigeria.

**Methods:**

We conducted a retrospective observational longitudinal evaluation of data that had been collected during the course of care in a large pediatric ART program in Nigeria. We used descriptive statistics to define our patient population and quantify retention from ART initiation through adolescence and transition to adult-centered care. Logistic regression was used to evaluate predictors of loss to follow-up. We used medication possession ratio (MPR) to quantify adherence for each year a patient was on ART. To evaluate adherence and virologic suppression, we measured the proportion of patients with ≥95% MPR and the proportion with virologic suppression (viral load ≤400 copies/mL) within each age cohort, and used bivariate analyses to examine any association between MPR and VL suppression for all person-years observed.

**Results:**

A total of 476 patients received at least one dose of ART as an adolescent (ages 10–19 years). The proportions of patients lost to follow-up were: 11.9% (71/597) prior to adolescence, 19.1% (31/162) during adolescence, and 13.7% (10/73) during transition to adult-centered care. While over 80% of patients had ≥95% medication adherence in all age groups, their viral load suppression rates through adolescence and post-transition were only 55.6%–64.0%. For patients that successfully transitioned to adult-centered care, we observed 87.7% (50/57) retention at month 12 post-transition, but only 34.6% (9/26) viral load suppression.

**Conclusions:**

Our evaluation found considerable proportions of adolescents lost to follow-up throughout the ART program cascade. We also found discrepancies between the proportions of patients with ≥95% MPR and the proportions with VL suppression, suggesting that true medication adherence in this population may be poor. Significant attention and targeted interventions to improve retention and adherence focused on adolescents are needed in order for global programs to achieve 90-90-90 goals.

## Introduction

With the great success of antiretroviral therapy (ART), larger numbers of children living with HIV are surviving into adolescence and adulthood. Adolescents, defined as persons aged 10–19 years, constitute a major driving force in the continued transmission of HIV globally, particularly in sub-Saharan Africa (SSA) [1]. Compared to a decade earlier, the global burden of adolescents living with HIV increased by 28%, largely attributed to perinatally infected children surviving into adolescence [2]. Additionally, while new infections among children aged 0–14 years declined by about 50% from 2009–2015, new infections among older adolescents aged 15–19 years declined by only 8% during the same period [2]. Modes of HIV acquisition during adolescence include transfusion, sexual transmission, and sharing of unsterile injection equipment. As of 2018, an estimated 1.6 million adolescents were living with HIV, with 80% in SSA [3]. Despite improved survival due to ART, AIDS is still the number one cause of death of adolescents in SSA and the second leading cause of death globally [1, 4].

From the start of the HIV epidemic, attention on adolescents living with HIV has been limited in many settings [4, 5]. Presently, scant empirical data exist on long-term outcomes in adolescent ART patients, particularly in resource-limited settings (RLS) [6–12]. Rates of adherence to treatment and retention in care are low for adolescents living with HIV [13, 14]. There is also a recognized gap in the published evidence on rates of transfer from child-centered care (CCC) to adult-centered care (ACC) and on subsequent outcomes following transfer, especially in RLS [15–17]. Whereas transfer refers to the movement of a child from CCC to ACC on a particular date or movement of a patient from one facility to another, transition can be defined as the purposeful and planned process preceding and following the transfer event during which the adolescent is assessed for readiness, prepared for transfer with knowledge and skills, and followed up after transfer to ensure retention and effective self-management of their health [18]. Most LMIC, including Nigeria, lack national guidelines for transition of adolescents in HIV care, and there is great in-country heterogeneity in transitional care models that vary by program [19, 20]. As more children living with HIV age into adolescence or become newly infected with HIV during adolescence, poor adherence, retention, and transfer rates could impede the continued progress made by global HIV programs. Research to quantify longitudinal trends and determinants of retention in adolescents and youth on ART is a critical foundation for developing targeted interventions to strengthen programs by improving adherence patterns, enhancing the transition process, and ensuring subsequent program retention [11].

We conducted a retrospective evaluation of routinely collected data from pediatric patients enrolled in the Harvard/AIDS Prevention Initiative in Nigeria (APIN) President’s Emergency Plan for AIDS Relief (PEPFAR) program between 2006 and 2016. We evaluated patient-level data to examine adherence, virologic suppression, and retention patterns through the adolescent years, the proportion that transitioned from CCC to ACC, and adherence, virologic suppression, and retention patterns 12 months post-transition.

## Methods

### Study design and participants

We conducted a retrospective evaluation of HIV-infected pediatric patients enrolled on ART between 2006 and 2016 at the Jos University Teaching Hospital (JUTH), a Harvard/APIN PEPFAR Program-supported ART site in Nigeria. The patients entered the program either following diagnosis of HIV infection through the prevention of mother-to-child transmission (PMTCT) clinics or through the HIV testing services. Upon enrollment in the Harvard/APIN PEPFAR-supported HIV care program, written informed consent for care was obtained from parents and written assent from children 7–17 years of age, both of which included an option for permission to use collected data for secondary research. All patients were assessed for ART eligibility according to age-specific Nigerian National ART Guidelines in place at the time the patient was under treatment, which followed the relevant current World Health Organization (WHO) recommendations at the time of receiving care and treatment [21, 22]. The data repository and consent forms were approved by the institutional review boards at Harvard, APIN, and JUTH, and this secondary analysis was determined exempt by the institutional review board at Harvard and non-human subjects research at APIN and JUTH.

All ART-eligible patients in the pediatric HIV program were placed on treatment by a pediatrician following a clinical examination and a set of baseline laboratory tests, which included hematology, clinical chemistries, CD4+ cell count, and plasma viral load (VL) measurements. The majority of patients were prescribed a 30-day supply of antiretroviral (ARV) medications. According to the clinical guidelines, following the first prescription pick-up, refills were obtained monthly and patients returned to the clinics every three months for the first year and every six months thereafter for routine clinical evaluation and laboratory monitoring, including hematology, clinical chemistries, CD4+ cell count, and VL, unless an earlier evaluation was medically indicated. In reality, some clinical and laboratory visits were late, missed, or not recorded; stock outs of test kits, i.e., VL assays, also resulted in missed laboratory tests. Patients with a failing VL result after 6 months of ART received enhanced adherence counseling, including assessment, education, and support, and were scheduled to return for a second VL test in three months. Those with a consecutive failing VL result were switched to second-line (2L) ART as determined by the pediatrician. All patient clinical, laboratory, and pharmacy data were entered and stored in electronic databases [23].

Per program protocol, adolescents receiving care in the JUTH pediatric HIV clinic transferred to the site’s adult HIV clinic, where they received new patient ID numbers, by 18 years, but as early as 16 years. The age of transfer depended on the adolescent’s readiness, willingness, and comfort, and on parents’ request (for some parents it was convenient to move their children earlier so that they could follow the same clinic schedule). The decision was made after an adherence counselor met with the adolescent and sometimes also with the parents/caregiver to discuss and assess the adolescent’s willingness, maturity, and understanding of their disease. We reviewed the clinical records of the adult HIV clinic to identify those who transitioned and to assess their 12-month outcomes. Additionally, we reviewed the records of 14 of JUTH’s PEPFAR-supported satellite HIV care sites in Plateau State to identify additional transfer patients that were not recorded in JUTH’s clinical records based on their demographic information. Additional transfer patients that were found at the pediatric satellite clinics were classified as transfers and censored on their last ART pick-up date at JUTH; no new transfer patients were found at the adult satellite clinics that were not already recorded at JUTH.

### Definitions

We evaluated patient variables at ART initiation (age, sex, HIV transmission risk category, enrollment year, ART regimen, and VL). Baseline clinical assessments or laboratory evaluations for naïve patients were the closest measurements to, and up to six months before or 0.5 months after, their first ART pick-up date. Virologic suppression was defined as a single VL below the limit of detection (≤400 cp/mL).

We classified patients as lost to follow-up (LTFU) if they had missed their last scheduled ART pick-up appointment by more than two months as of the date the study data were censored. Pediatric program data were censored on July 29, 2016; data for patients who transferred to the adult program were censored at 12 months after the date of transfer (up to July 29, 2017). Patients who missed a scheduled appointment by more than two months, but returned to the clinic before the data censor date, were classified as retained in care; their treatment lapse would be indicated in their adherence measurement. Patients for whom death, withdrawal, or transfer to non-Harvard/APIN sites was recorded during the period of evaluation were not considered LTFU. Data on deaths, withdrawals, or transfers were passively obtained when either patients or their parents/caregivers provided the information; active tracking of patients that were late to appointments was not feasible as part of the program.

We used medication possession ratio (MPR), as a surrogate for adherence, to quantify adherence patterns [24–27]. For each complete year of age that a patient remained on ART, we computed MPR for that year as the total number of pills provided during that year divided by 365 days and then multiplied by 100; if a patient discontinued ART for any reason or was censored during a given year, the denominator was the number of days the patient remained on ART during that year. We stratified these average percent adherence values into clinically relevant categories for analyses: optimal (≥95%), suboptimal (80%–94%), and poor (<80%), based on previously established thresholds.

### Statistical analysis

We summarized baseline demographics and clinical characteristics using standard descriptive statistical methods. Additionally, we plotted patient entry and exit points from the cohort to demonstrate numbers of patients included in the evaluation, highlighting those that entered prior to adolescence (i.e., <10 years) and those that entered during adolescence (i.e., age 10–19 years). Bivariate comparisons of categorical variables were performed using the *Χ*^2^ test and Fisher’s exact test, as relevant, and of continuous variables using Student’s t-test and the Wilcoxon rank sum test, as relevant. Statistical significance was defined at an α-level of 0.05.

We measured retention in CCC as a proportion: the number of adolescents (≥10 years) in the cohort receiving care at the age of transition (≥16 years) or at the censor date over the number of adolescents who received at least one dose of ART. Similarly, we measured retention in ACC as the proportion of patients receiving care at 12 months in ACC over the number who transitioned to ACC and had 12 months of observation time (not censored). Patients who transferred outside of JUTH’s PEPFAR network were treated as censored on the date of transfer for the analyses.

We built a logistic regression model to examine predictors of LTFU. We considered all variables that were significant at the α-level of 0.20 in bivariate evaluations for the multivariate model. Potential relevant interaction terms were also tested as part of the modeling process, but no significant interactions were found at the α-level of 0.05. We excluded potential predictors that were not significant in the multivariate model by stepwise backward selection, and presented only variables that remained significant at the α-level of 0.05 in the adjusted model.

To examine adherence as measured by MPR and VL suppression patterns by age, we conducted small cross-sectional evaluations by creating cohorts by age, including children in a group if they received treatment for at least two months during that year. If a patient had more than one VL during a given year, we used first measurement for the analyses. For each age cohort, we computed the proportion of patients with ≥95% adherence as measured by MPR and the proportion of patients with suppressed VL. We computed proportions with ≥95% MPR for all patients in the age cohort as well as for only those with VL values. We also looked at all person-years with VL values and calculated an overall proportion with ≥95% MPR and an overall proportion with suppressed VL, and performed bivariate analysis to examine any association between MPR and VL suppression.

As a sensitivity analysis for patients who dropped out part way through a year due to either death, withdrawal or LTFU, we also computed MPR values for age cohorts using an intent-to-treat (ITT) approach, where MPR for the drop out year was calculated as number of pills provided during that year until drop out divided by the full 365 days. There was no statistically significant difference between the proportions of patients with ≥95% adherence as measured by MPR using the per protocol (PP) and ITT adherence values; thus, we reported PP data. As a sensitivity analysis for missing VL data, multiple imputations of VL data for each age category were generated and we recomputed and compared the proportions with undetectable VL, and no difference was found; therefore, we have presented values based on those patients with data available. Further, we performed another sensitivity analysis for missing VL data in which we considered patients with missing VL as detectable and repeated the bivariate analysis to evaluate any association between MPR and VL suppression.

We conducted all statistical analyses using Stata version 13.1 (Stata Corporation, College Station, Texas, USA).

## Results

**Fig 1:**
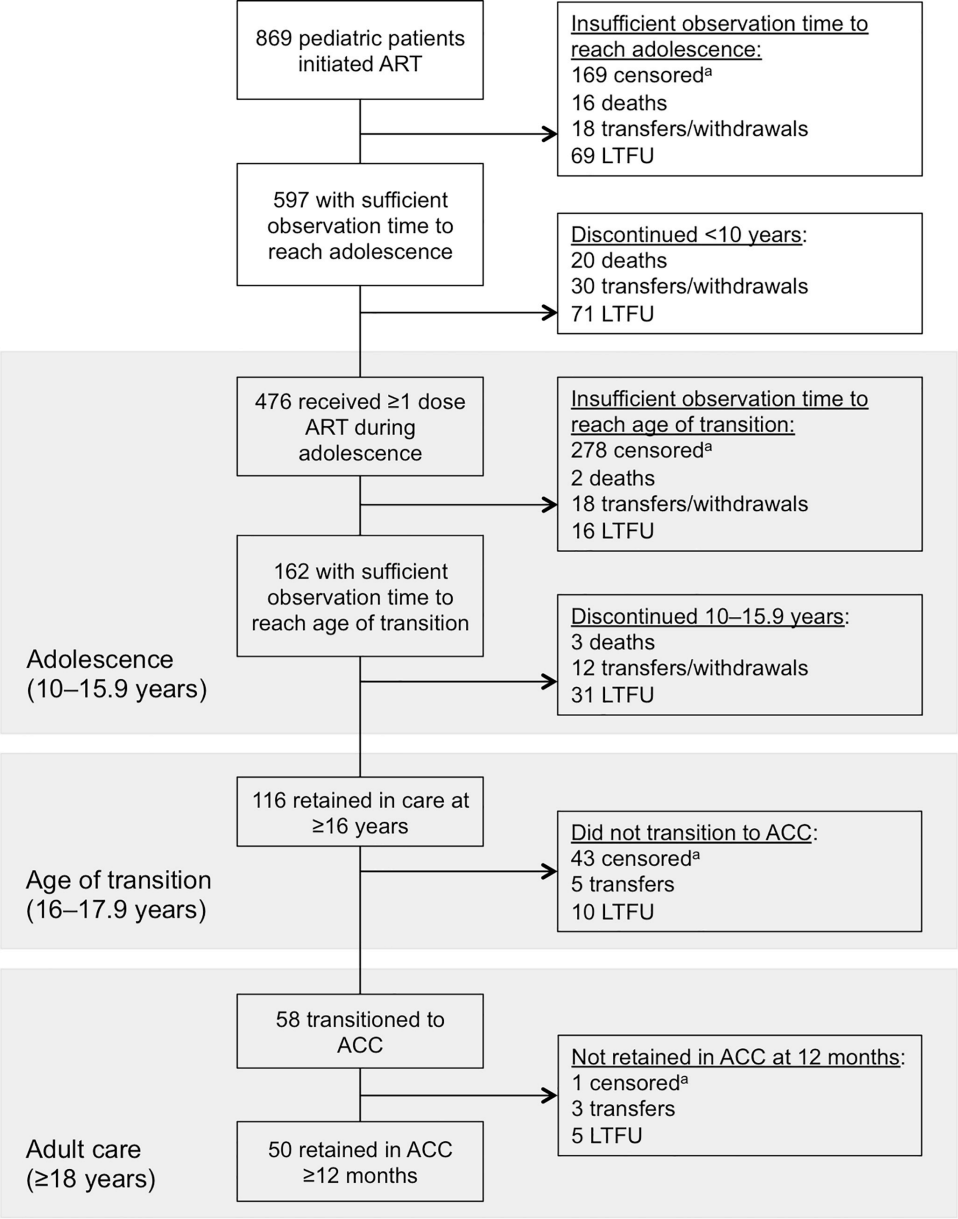
Flow chart of patients. ART, antiretroviral therapy; LTFU, loss to follow-up; JUTH, Jos University Teaching Hospital; CCC, child-centered care; ACC, adult-centered care. ^a^Censored due to reaching end of study period.

A total of 869 children initiated ART at JUTH between February 2006 and July 2016 (see S1 Fig, which represents a longitudinal overview of pediatric patient entry and retention). Of those patients, 597 (68.7%) had entry dates that would have allowed them to reach the age of adolescence (i.e., 10 years) during the observation period if retained on treatment. Out of those eligible by entry date to reach adolescence by the end of the data collection period, 476 (79.7%) received at least one dose of ART as an adolescent, while 71 (11.9%) were considered LTFU (Fig 1).

**Fig 2:**
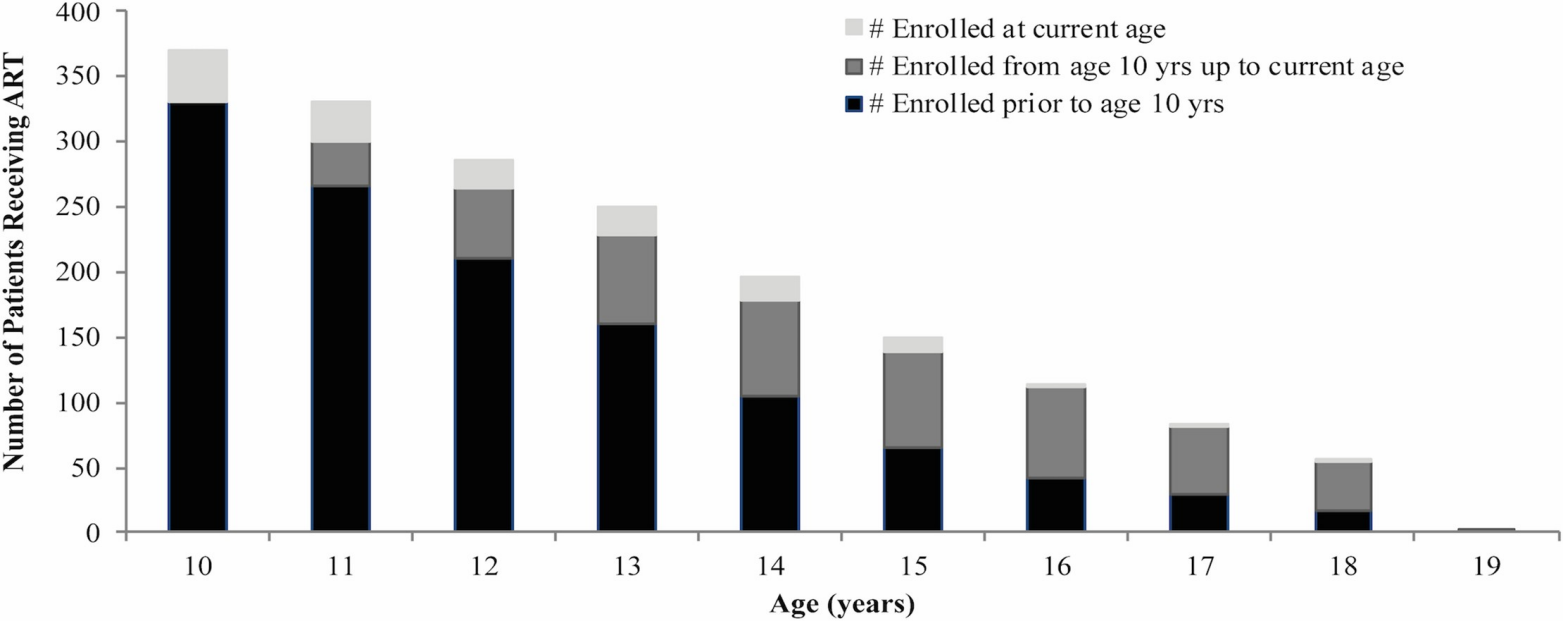
Number of patients that received treatment during adolescent years by age, stratified by age at ART initiation.

After creating cross-sectional age cohorts for each adolescent year from age 10 to 19, we found that the largest age cohort within the adolescent group was those aged 10 years (Fig 2). The majority (n = 331; 69.5%) of children that received ART during their adolescent years, initiated ART prior to age 10 years and the remaining adolescents, initiated ART in the program at age 10 years or higher (n = 145; 30.5%).

Of the adolescents that initiated ART prior to age 10 years, 304 (91.8%) were infected through MTCT, whereas of those that entered at age 10 years or above, 91 (62.7%) acquired HIV through MTCT; for the children initiating ART at age 10 years or above, 42 (29.0%) did not have a documented mode of transmission (Table 1). The median age at enrollment for all adolescents evaluated in this analysis was 4.3 years (interquartile range (IQR): 0.8–7.3) and the median time on treatment in the program for all adolescents was 7.2 years (IQR: 3.6–8.9). The cohort was 50.8% (n = 242) female, with the majority of those initiating at ≥10 years being female (60.0%). Over half (52.5%) of the children in the cohort were initiated on ART regimens containing zidovudine + lamivudine + nevirapine.

**Table 1:** Demographic characteristics of adolescent population, by period of program enrollment.

Characteristics	All Adolescents (10–19 years)	Initiated ART at age <10 years	Initiated ART at age ≥10 years
**Number**	476	331	145
**Sex, n (%)**			
Female	242 (50.8)	155 (46.8)	87 (60.0)
Male	234 (49.2)	176 (53.2)	58 (40.0)
**ART initiation year, n (%)**			
2006	113 (23.7)	90 (27.2)	23 (15.9)
2007	118 (24.8)	98 (29.6)	20 (13.8)
2008	65 (13.7)	51 (15.4)	14 (9.7)
2009	43 (13.7)	30 (9.1)	13 (9.0)
2010	16 (3.4)	12 (3.6)	4 (2.8)
2011	26 (5.5)	16 (4.8)	10 (6.9)
2012	37 (7.8)	21 (6.3)	16 (11.0)
2013	25 (5.3)	8 (2.4)	17 (11.7)
2014	11 (2.3)	3 (0.9)	8 (5.5)
2015	18 (3.8)	2 (0.6)	16 (11.0)
2016	4 (0.8)	0 (0.0)	4 (2.8)
**Previous ARV experience, n (%)**	61 (12.8)	33 (10.0)	28 (19.3)
**Median age at entry, years (IQR)**	4.3 (0.8–7.3)	3.2 (0.8–5.4)	10.9 (10.0–12.2)
**Median time on treatment, years (IQR)**	7.2 (3.6–8.9)	8.4 (6.1–9.3)	3.0 (1.3–4.6)
**Median time on treatment as adolescent, year (IQR)**	2.9 (1.2–4.6)	2.7 (1.2–4.6)	3.1 (1.2–4.6)
**Main transmission mode, n (%)**			
Mother-to-child	395 (83.0)	304 (91.8)	91 (62.7)
Blood transfusion	15 (3.2)	6 (1.8)	9 (6.2)
Needle sharing	1 (0.2)	0 (0.0)	1 (0.7)
Sexual abuse/rape	2 (0.4)	1 (0.3)	1 (0.7)
Heterosexual transmission	1 (0.2)	0 (0.0)	1 (0.7)
Unknown	62 (13.0)	20 (6.0)	42 (29.0)
**ART regimen at initiation, n (%)**			
AZT+3TC+NVP	250 (52.5)	175 (52.9)	75 (51.7)
AZT+3TC+EFV	151 (31.7)	107 (32.3)	44 (30.3)
Other	75 (15.8)	49 (14.8)	26 (17.9)
**Median log VL at ART initiation, copies/mL (IQR)**	4.72 (4.20–5.36)	4.63 (4.09–5.32)	4.94 (4.31–5.42)

ART, antiretroviral therapy; ARV, antiretroviral; IQR, interquartile range; AZT, zidovudine; 3TC, lamivudine; NVP, nevirapine; EFV, efavirenz; VL, viral load.

### Retention and loss to follow-up in adolescents

Overall, 379 of 476 (79.6%) of the adolescents that received ART were retained in CCC or transitioned to ACC at the end of the study period. Of those, 283 (74.7%) had been enrolled prior to adolescence while the remaining patients were newly initiated on ART during adolescence. 97 (20.4%) adolescent ART patients were not retained prior to reaching age 18 years; 5 (1.1%) had died, 35 (7.4%) transferred or withdrew, and 57 (12.0%) were considered LTFU. Within the LTFU group, 24 (42.1%) had enrolled on ART prior to age 10 years and 32 (56.1%) enrolled during adolescence. In multivariate logistic modeling controlling for potential confounding variables (sex, previous ARV experience, first-line ART containing zidovudine + lamivudine vs. other, and transmission via mother-to-child vs. other), older age at ART initiation, later enrollment year, and longer duration on ART appeared to have slightly lower odds of LTFU (Table 2).

**Table 2:** Predictors of loss to follow-up from child-centered care among adolescent patients on antiretroviral therapy.

Predictor	Unadjusted OR (95% CI)	p-value	Adjusted OR (95% CI)
Age at ART initiation, years	1.21 (1.12–1.30)	<0.001	0.72 (0.61–0.86)
ART initiation prior to age 10 years	0.27 (0.15–0.47)	<0.001	
Female sex	1.10 (0.63–1.92)	0.74	
Previous ARV experience	2.02 (0.99–4.12)	0.052	
1L NRTI AZT+3TC vs Other	0.23 (0.11–0.50)	<0.001	
Transmission via MTCT	0.41 (0.22–0.77)	0.006	
Enrollment year	0.95 (0.85–1.06)	0.33	0.51 (0.42–0.62)
Duration on ART, years	0.999 (0.998–0.999)	<0.001	0.997 (0.996–0.998)

OR, odds ratio; CI, confidence interval; ART, antiretroviral therapy; ARV, antiretroviral; 1L, first-line; NRTI, nucleoside reverse transcriptase inhibitor; AZT, zidovudine; 3TC, lamivudine; MTCT, mother-to-child transmission.

### Viral load suppression and adherence

Of the total person-years (1,801 person-years), the proportion with ≥95% adherence as measured by MPR was 85.0%, and of those that had VL data (906 person-years), the proportion with ≥95% MPR was 83.6%. However, the proportion of total person-years with suppressed VL was 61.0%.

**Fig 3:**
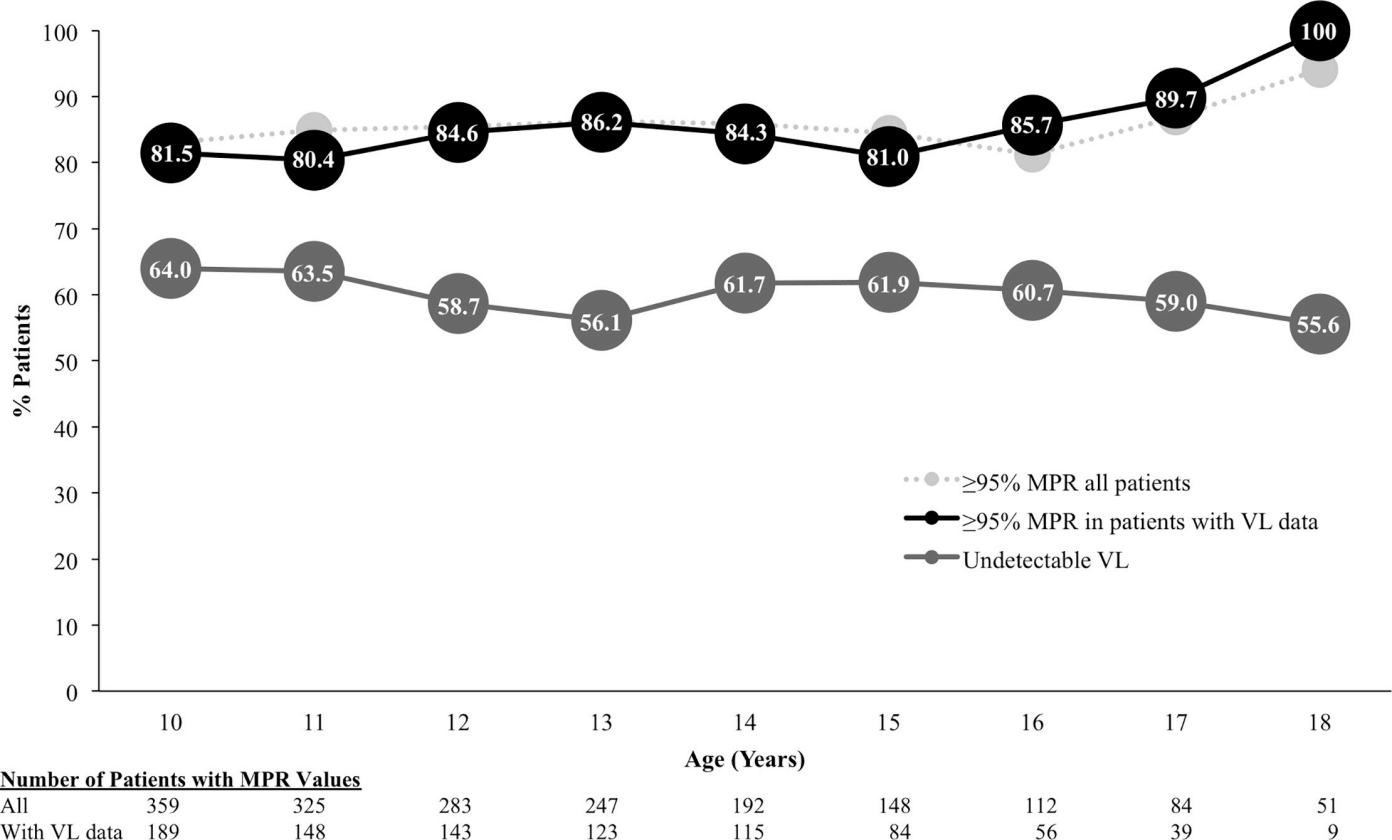
Adherence as measured by MPR and VL during adolescent years by age cohorts. MPR, medication possession ratio; VL, viral load.

Within each age cohort, the proportion of adolescents with follow-up VL values and average adherence ≥95%, as measured by MPR, ranged from 80.4% to 100% for all age cohorts (Fig 3). Of note, the proportion of adolescents with ≥95% MPR did not vary significantly by age cohort, regardless of VL data availability. As we observed overall, higher proportions with optimal adherence as measured by MPR did not appear to translate into higher proportions with undetectable VL within each age cohort; for patients with VL data, we computed proportion with undetectable follow-up VL by age and found that proportion of patients with suppressed VL ranged from 55.6% to 64.0% during the adolescent years.

In bivariate analyses, examining association between adherence as measured by MPR and VL for all person-years where patients had VL data, we found that patients with ≥95% MPR were more likely than those with <95% MPR to have undetectable VL. 64.5% of the person-years for patients with ≥95% MPR were associated with undetectable VL whereas 44.2% of person-years for patients with <95% MPR were associated with undetectable VL (p<0.001). While optimal MPR was positively correlated with VL suppression, the proportions with undetectable VL were low among both those with optimal and sub-optimal MPR.

The number of missing VLs over all person-years was considerable; 49.7% (895/1,801) of total person-years were missing VLs. However, we did not see a significant difference in MPR between those with missing VLs versus those with VLs. As a sensitivity analysis for person-years that were missing VLs, we performed another bivariate analysis in which we considered all missing VLs detectable; we still found a significant correlation between MPR and VL suppression, with 31.8% of person-years for patients with ≥95% MPR were associated with undetectable VL compared to 24.6% of the person-years for patients with <95% MPR were associated with undetectable VL (p = 0.015).

### Transition from pediatric to adult program

For the evaluation of transition from CCC to ACC, a total of 162 patients that received at least one dose of ART as adolescents had enough observation time from their ART initiation point to reach age ≥16 years. Of the patients that had sufficient time since entry to have transitioned to ACC, 116 (71.6%) reached age 16 years while still in the JUTH ART program. The remaining patients did not reach age of transition in the JUTH program, where 12 (7.4%) transferred or withdrew prior to or at the time of reaching transition age, 3 (1.9%) had died, and 31 (19.1%) were LTFU. Of the 116 patients who reached age 16 years in the JUTH program, 43 (26.5%) had yet to transition at the end of the cohort observation period. Of the remaining 73 patients not censored, 58 (79.5%) were transitioned to ACC and 10 (13.7%) were LTFU. The median age at ART initiation for the patients for whom we could evaluate transition was 11.2 years (IQR: 9.5–13.9 years) and of those patients, 17 (29.3%) had initiated ART prior to age 10 years.

Of the 58 patients that transitioned, 57 (98.3%) had been enrolled with sufficient observation time to evaluate retention at 12 months in ACC; of those patients, 50 (87.7%) were retained for at least 12 months post-transition, and 5 (8.8%) were LTFU. Despite 87.7% retention at 12 months post-transition, VL data were only available for 26 of those retained; of those with VL data, only 9 (34.6%) had an undetectable VL at 12 months post-transition. A sensitivity analysis comparing the 26 patients who had VL results and the 24 without VL results at month 12 showed that patients with risk factors other than MTCT or unknown appeared more likely to have VL results (70.6%) than those who acquired HIV through MTCT (42.4%, p = 0.059); no differences were seen between the two groups by sex, age at enrollment, previous ARV experience, enrollment year, duration on ART, regimen, or VL at time of transition to adult program.

## Discussion

Our retrospective evaluation found that 19.1% (31/162) of adolescents who received ART in the pediatric program and had enough observation time to reach age ≥16 years were LTFU before reaching the age of transition. Furthermore, while we found that over 80% of adolescents had ≥95% MPR in all age groups, less than 64.0% had VL suppression, suggesting that true adherence to medication was poor. Achieving high levels of retention and adherence in adolescent populations is fraught with challenges, which include stigma and discrimination, concomitant substance abuse, forgetfulness, suspicion of therapy, complicated regimens, decreased quality of life, work and family responsibilities, non-disclosure of HIV status, pill burden, drug toxicity and cost of transportation to the health facility [28]. The unique psychosocial and developmental issues facing youth are more varied and dynamic than those of adults, as they must simultaneously deal with additional adolescent concerns including issues with body image, first sexual experience, mental health issues, peer pressure and identity formation [29–34]. Additionally, compliance with drug regimens oftentimes worsens as a child becomes more independent from parental control [35]. Anecdotally, our program clinicians note that a related issue in the Nigerian setting is school attendance; while children typically live with their parents during their primary school years, some shift to boarding schools as adolescents, where supervision of adherence and retention as well as social stigma present more challenges [36, 37].

Beyond adolescence, another consideration regarding long-term retention is transition to ACC. In our program, we found that 13.7% (10/73) of patients who reached the age of transfer and had sufficient observation time to transition to ACC were LTFU. More research is needed to identify the reasons why some adolescents fail to transition in order to inform program-specific interventions that promote successful transition [6, 11, 30, 38]. Although facilitators of transition have been indicated through research on other congenital conditions and HIV programs in resource-rich settings, such as use of guided transition teams, peer groups, and development of adolescent clinics, there are still insufficient data on factors that optimize transfer and subsequent HIV retention rates in RLS despite a growing body of recent research [9, 39–44]. Recent qualitative research has been aiming to identify both barriers and facilitators of successful transition, leading to recommendations that include development of surveillance systems for monitoring adolescents during transition, development of transition guidelines and policies, multidisciplinary clinic models, dedicated transition staff, peer support groups, training of adult providers, and communication between pediatric and adult clinics [45, 46]. Pettitt ED et al. have reported that successful transition to ACC can be promoted by a comprehensive, adolescent-friendly care model that includes support for their psychosocial needs and requires increased collaboration among healthcare providers [7]. In Nigeria, the Adolescent Coordinated Transition (ACT) two-arm randomized control trial is currently being conducted to compare retention and VL suppression with a graduated transition and organized support group intervention against the standard practices used in transferring adolescents from pediatric to adult care [47]. Overall, retention through the adolescent period through transition to ACC will require different types of support, which change with time and child development [48–50]. Interestingly, we found that in the JUTH program, of those that transitioned to ACC, 87.7% (50/57) were retained for at least 12 months post-transition, suggesting that a high level of retention is possible following a successful transition.

In this longitudinal evaluation, the proportion of patients with VL suppression throughout the adolescent years was below 64.0% among those with VL data. VL suppression was especially poor among the patients that were retained on ART at least 12 months post-transition to ACC, at a mere 34.6% (9/26) among those with VL data. This poor VL suppression in adolescents was most concerning of our findings in this evaluation. In the era of 90-90-90, where the third goal envisions 90% of patients virally suppressed, adolescents will pose a particular challenge in achieving targets.

Prior studies utilizing pharmacy refill and/or MPR as proxy measures of adherence in adults have shown strong correlation with clinical outcomes [25–27, 51]. However, in this evaluation we found that high MPR did not correspond with high proportions of VL suppression for all age strata through the adolescent years. One possible explanation for this incongruity is suboptimal adherence. It is possible these children or their guardians are obtaining the medications, but the adolescents are either not taking the medications or not correctly taking their full regimens. The psychosocial and developmental issues faced during adolescence may present as barriers to adherence, and adolescents may be prone to hiding their poor adherence due to fear of judgment or punishment by clinicians, counselors, or the guardians who accompany them to pick up their medications. Another possible explanation for the poor correlation between MPR and VL suppression is drug resistance. With median time on treatment of 7.2 years, the long duration on ART for these adolescents increases their risk for developing drug resistance mutations. Despite current adherence, drug resistance will lead to VL rebound and persistent non-suppression if the patient is not switched to an effective 2L regimen in a timely manner, per program protocol. In some other study settings, pill counts have been used as a proxy measure for adherence. A Botswana study found that adolescents in virologic failure were more likely than those with viral suppression to have their adherence as measured by pill count consistently >100%. Adolescents may attempt to hide their poor adherence by ‘pill dumping’, or discarding pills to make it appear they were consumed [52]. The disparity between proxy measurements of adherence like MPR and pill counts and viral suppression indicates discord with true adherence rates.

Our findings suggest that both adherence counseling and monitoring for adolescents may require different methods than what is typically used for adults. Adherence counseling for adolescents should be effective without feeling punitive such that some adolescents may feel a need to hide poor adherence. Furthermore, since monitoring MPR does not necessarily provide a clear picture of VL outcomes, more regular VL monitoring for adolescents might be warranted, and particularly during the first year after transition to ACC. This will enable more timely adherence interventions, drug resistance testing when criteria are met, and quicker switch to 2L. Considering the widening disparity in MPR and VL suppression for 17 and 18 year olds, we suggest routine drug susceptibility testing for all viremic adolescents at the time of transition to ACC where resources are available. More research is required to clarify the potential clinical, psychosocial, behavioral and logistical reasons behind the disparity between the measures and to develop better ways to monitor and support adolescents.

To our knowledge, this is the first longitudinal evaluation of a pediatric ART cohort through transition to adult care in Nigeria. A strength of this study is that we have over ten years of observational data, including available VL measurements, through which we were able to track the patients through adolescence and transition from CCC to ACC. A notable limitation given our study design was a small sample size for assessing transition from CCC to ACC. It also must be noted that the number of missing VLs was considerable. However, we found no significant difference in MPR between those with missing VLs versus those with no missing VLs and were able to demonstrate no change in overall interpretation of results in our sensitivity analyses. The lack of association between MPR and VL missingness may suggest that the missing VLs were due to programmatic issues, i.e., test kit stock outs, rather than patient level of engagement in care. It is also possible that clinicians may be more likely to prioritize VL testing for patients who appear to be less adherent or are not doing well clinically. Overall, despite the limitations, our study design allowed us to track a single cohort and show that even with limited numbers, we could reinforce that retention in adolescence must be addressed, and that for those that successfully transitioned and were retained 12 months post-transition, virologic suppression is a critical issue and adherence must be effectively measured and reinforced.

## Conclusions

There remains a need for research to inform development of evidence-based adolescent adherence and retention interventions as well as transition models for ART programs in RLS. As determinants of retention might differ by population, understanding rates of transfer and influences on those rates in RLS are critical for building evidence-based HIV-specific health care transition models in these settings [53]. Our goal in presenting empirical data spanning 10 years was to provide evidence for the need for formal interventions aimed at improving outcomes—retention, adherence, and VL suppression—of adolescents living with HIV transitioning from pediatric to adult ART programs. We found considerable proportions of adolescents LTFU throughout the ART program cascade. While adherence rates measured by MPR appeared high, they did not correspond with proportions of patients with VL suppression, which were alarmingly low. For patients that transitioned, the proportion retained after a year was high, but VL outcomes poor. Further investigation will be necessary to determine the reasons for LTFU and poor VL suppression in this key population group and design appropriate interventions to ensure that no group is left behind as we work towards attainment of the 90-90-90 targets.

## Supporting information

S1 Fig Longitudinal overview of pediatric patient entry and retention (n = 869). Shaded boxes highlight patients that received ART as adolescents; green shading highlights patients that initiated ART prior to adolescence and yellow shading highlights patients that initiated ART as adolescents.
(TIF)

## References

[1] WHO. Health for the world's adolescents: A second chance in the second decade. Geneva: World Health Organization; 2014.

[2] UNICEF. For every child end AIDS, seventh stocktaking report. New York: UNICEF; 2016.

[3] UNAIDS. AIDSinfo: Indicators: People living with HIV. UNAIDS AIDSinfo website [Available from: http://aidsinfo.unaids.org/.

[4] UNICEF. ALL IN! to end adolescent AIDS: A progress report. New York; 2016.

[5] Idele P, Gillespie A, Porth T, Suzuki C, Mahy M, Kasedde S, et al Epidemiology of HIV and AIDS among adolescents: current status, inequities, and data gaps. J Acquir Immune Defic Syndr. 2014;66 Suppl 2:S144–53. 24918590 10.1097/QAI.0000000000000176

[6] Cervia JS. Easing the transition of HIV-infected adolescents to adult care. AIDS Patient Care STDS. 2013;27(12):692–6. 10.1089/apc.2013.0253 24073595 PMC3868277

[7] Pettitt ED, Greifinger RC, Phelps BR, Bowsky SJ. Improving health services for adolescents living with HIV in sub-Saharan Africa: a multi-country assessment. Afr J Reprod Health. 2013;17(4 Spec No):17–31. 24689314

[8] Lee S, Hazra R. Achieving 90-90-90 in paediatric HIV: adolescence as the touchstone for transition success. J Int AIDS Soc. 2015;18(7 Suppl 6):20257. 26639113 10.7448/IAS.18.7.20257 PMC4670843

[9] Phelps BR, Ahmed S, Amzel A, Diallo MO, Jacobs T, Kellerman SE, et al Linkage, initiation and retention of children in the antiretroviral therapy cascade: an overview. AIDS. 2013;27 Suppl 2:S207–13. 24361630 10.1097/QAD.0000000000000095 PMC4124132

[10] Tulloch O, Theobald S, Ananworanich J, Chasombat S, Kosalaraksa P, Jirawattanapisal T, et al From transmission to transition: lessons learnt from the Thai paediatric antiretroviral programme. PLoS One. 2014;9(6):e99061 10.1371/journal.pone.0099061 24893160 PMC4043947

[11] Mofenson LM, Cotton MF. The challenges of success: adolescents with perinatal HIV infection. J Int AIDS Soc. 2013;16:18650 10.7448/IAS.16.1.18650 23782484 PMC3687076

[12] Evans D, Menezes C, Mahomed K, Macdonald P, Untiedt S, Levin L, et al Treatment outcomes of HIV-infected adolescents attending public-sector HIV clinics across Gauteng and Mpumalanga, South Africa. AIDS Res Hum Retroviruses. 2013;29(6):892–900. 10.1089/AID.2012.0215 23373540 PMC3653371

[13] Agwu AL, Lee L, Fleishman JA, Voss C, Yehia BR, Althoff KN, et al Aging and loss to follow-up among youth living with human immunodeficiency virus in the HIV Research Network. J Adolesc Health. 2015;56(3):345–51. 10.1016/j.jadohealth.2014.11.009 25703322 PMC4378241

[14] Meloni ST, Chaplin B, Chang C, Rawizza H, Okonkwo P, Kanki PJ. Patterns of Adherence and Loss to Follow-Up in Pediatric Patients on ART in Nigeria. Curr HIV Res. 2015;13(3):210–8. 10.2174/1570162x1303150506183921 25986372

[15] Reisner SL, Mimiaga MJ, Skeer M, Perkovich B, Johnson CV, Safren SA. A review of HIV antiretroviral adherence and intervention studies among HIV-infected youth. Top HIV Med. 2009;17(1):14–25. 19270345 PMC3752381

[16] Hussen SA, Chahroudi A, Boylan A, Camacho-Gonzalez AF, Hackett S, Chakraborty R. Transition of youth living with HIV from pediatric to adult-oriented healthcare: a review of the literature. Future Virol. 2015;9(10):921–9. 10.2217/fvl.14.73 25983853 PMC4433446

[17] Judd A, Davies MA. Adolescent transition among young people with perinatal HIV in high-income and low-income settings. Curr Opin HIV AIDS. 2018;13(3):236–48. 10.1097/COH.0000000000000448 29528851 PMC6424353

[18] Njuguna I, Beima-Sofie K, Mburu C, Mugo C, Black DA, Neary J, et al Managing the transition from paediatric to adult care for HIV, Kenya. Bull World Health Organ. 2019;97(12):837–45. 10.2471/BLT.19.232702 31819292 PMC6883269

[19] Dahourou DL, Gautier-Lafaye C, Teasdale CA, Renner L, Yotebieng M, Desmonde S, et al Transition from paediatric to adult care of adolescents living with HIV in sub-Saharan Africa: challenges, youth-friendly models, and outcomes. J Int AIDS Soc. 2017;20(Suppl 3):21528 10.7448/IAS.20.4.21528 28530039 PMC5577723

[20] Badejo OA, Menson WNA, Sam-Agudu NA, Pharr J, Erekaha S, Bruno T, et al Pediatric to adult healthcare transitioning for adolescents living with HIV in Nigeria: A national survey. PLoS One. 2018;13(6):e0198802 10.1371/journal.pone.0198802 29894519 PMC5997346

[21] Nigeria FMoH. National guidelines for HIV and AIDS treatment and care in adolescents and adults. Abuja, Nigeria; 2007.

[22] Nigeria FMoH. National guidelines for HIV and AIDS treatment and care in adolescents and adults. Abuja, Nigeria; 2010.

[23] Chaplin B, Meloni S, Eisen G, Jolayemi T, Banigbe B, Adeola J, et al Scale-up of networked HIV treatment in Nigeria: creation of an integrated electronic medical records system. Int J Med Inform. 2015;84(1):58–68. 10.1016/j.ijmedinf.2014.09.006 25301692

[24] Meloni ST, Chang CA, Eisen G, Jolayemi T, Banigbe B, Okonkwo PI, et al Long-Term Outcomes on Antiretroviral Therapy in a Large Scale-Up Program in Nigeria. PLoS One. 2016;11(10):e0164030 10.1371/journal.pone.0164030 27764094 PMC5072640

[25] Goldman JD, Cantrell RA, Mulenga LB, Tambatamba BC, Reid SE, Levy JW, et al Simple adherence assessments to predict virologic failure among HIV-infected adults with discordant immunologic and clinical responses to antiretroviral therapy. AIDS Res Hum Retroviruses. 2008;24(8):1031–5. 10.1089/aid.2008.0035 18724803 PMC2747786

[26] Low-Beer S, Yip B, O'Shaughnessy MV, Hogg RS, Montaner JS. Adherence to triple therapy and viral load response. J Acquir Immune Defic Syndr. 2000;23(4):360–1. 10.1097/00126334-200004010-00016 10836763

[27] Nachega JB, Hislop M, Dowdy DW, Lo M, Omer SB, Regensberg L, et al Adherence to highly active antiretroviral therapy assessed by pharmacy claims predicts survival in HIV-infected South African adults. J Acquir Immune Defic Syndr. 2006;43(1):78–84. 10.1097/01.qai.0000225015.43266.46 16878045

[28] Mills EJ, Nachega JB, Buchan I, Orbinski J, Attaran A, Singh S, et al Adherence to antiretroviral therapy in sub-Saharan Africa and North America: a meta-analysis. JAMA. 2006;296(6):679–90. 10.1001/jama.296.6.679 16896111

[29] Petersen I, Bhana A, Myeza N, Alicea S, John S, Holst H, et al Psychosocial challenges and protective influences for socio-emotional coping of HIV+ adolescents in South Africa: a qualitative investigation. AIDS Care. 2010;22(8):970–8. 10.1080/09540121003623693 20229370 PMC3037257

[30] Hazra R, Siberry GK, Mofenson LM. Growing up with HIV: children, adolescents, and young adults with perinatally acquired HIV infection. Annu Rev Med. 2010;61:169–85. 10.1146/annurev.med.050108.151127 19622036

[31] Mellins CA, Tassiopoulos K, Malee K, Moscicki AB, Patton D, Smith R, et al Behavioral health risks in perinatally HIV-exposed youth: co-occurrence of sexual and drug use behavior, mental health problems, and nonadherence to antiretroviral treatment. AIDS Patient Care STDS. 2011;25(7):413–22. 10.1089/apc.2011.0025 21992620 PMC3125549

[32] MacDonell K, Naar-King S, Huszti H, Belzer M. Barriers to medication adherence in behaviorally and perinatally infected youth living with HIV. AIDS Behav. 2013;17(1):86–93. 10.1007/s10461-012-0364-1 23142855 PMC3549030

[33] Martinez J, Chakraborty R. Psychosocial support for youth living with HIV. Pediatrics. 2014;133(3):558–62. 10.1542/peds.2013-4061 24567016

[34] Lowenthal ED, Bakeera-Kitaka S, Marukutira T, Chapman J, Goldrath K, Ferrand RA. Perinatally acquired HIV infection in adolescents from sub-Saharan Africa: a review of emerging challenges. Lancet Infect Dis. 2014;14(7):627–39. 10.1016/S1473-3099(13)70363-3 24406145 PMC4074242

[35] Bangsberg DR. Factors impacting adherence. In: Jani A, editor. Adherence to antiretroviral treatment regimens: recommendations for best practice. Washington, D.C.: APHA; 2004 p. 20–5.

[36] Mutwa PR, Van Nuil JI, Asiimwe-Kateera B, Kestelyn E, Vyankandondera J, Pool R, et al Living situation affects adherence to combination antiretroviral therapy in HIV-infected adolescents in Rwanda: a qualitative study. PLoS One. 2013;8(4):e60073 10.1371/journal.pone.0060073 23573232 PMC3616046

[37] Madiba S, Josiah U. Perceived Stigma and Fear of Unintended Disclosure are Barriers in Medication Adherence in Adolescents with Perinatal HIV in Botswana: A Qualitative Study. Biomed Res Int. 2019;2019:9623159 10.1155/2019/9623159 31886271 PMC6914939

[38] Bain-Brickley D, Butler LM, Kennedy GE, Rutherford GW. Interventions to improve adherence to antiretroviral therapy in children with HIV infection. Cochrane Database Syst Rev. 2011(12):CD009513 10.1002/14651858.CD009513 22161452 PMC6599820

[39] Crowley R, Wolfe I, McKee M. Improving the transition between paediatric and adult healthcare: a systematic review. Arch Dis Child. 2011;96(6):548–53. 10.1136/adc.2010.202473 21388969

[40] Bundock H, Fidler S, Clarke S, Holmes-Walker DJ, Farrell K, McDonald S, et al Crossing the divide: transition care services for young people with HIV-their views. AIDS Patient Care STDS. 2011;25(8):465–73. 10.1089/apc.2010.0279 21745141

[41] Lugasi T, Achille M, Stevenson M. Patients' perspective on factors that facilitate transition from child-centered to adult-centered health care: a theory integrated metasummary of quantitative and qualitative studies. J Adolesc Health. 2011;48(5):429–40. 10.1016/j.jadohealth.2010.10.016 21501800

[42] Wiener LS, Zobel M, Battles H, Ryder C. Transition from a pediatric HIV intramural clinical research program to adolescent and adult community-based care services:assessing transition readiness. Soc Work Health Care. 2007;46(1):1–19. 10.1300/J010v46n02_01 18032153 PMC2366035

[43] Newman C, Persson A, Miller A, Cama E. Bridging worlds, breaking rules: Clinician perspectives on transitioning young people with perinatally acquired HIV into adult care in a low prevalence setting. AIDS Patient Care STDS. 2014;28(7):381–93. 10.1089/apc.2013.0346 24749770

[44] Andiman WA. Transition from pediatric to adult healthcare services for young adults with chronic illnesses: the special case of human immunodeficiency virus infection. J Pediatr. 2011;159(5):714–9. 10.1016/j.jpeds.2011.06.040 21868035

[45] Straub DM, Tanner AE. Health-care transition from adolescent to adult services for young people with HIV. Lancet Child Adolesc Health. 2018;2(3):214–22. 10.1016/S2352-4642(18)30005-1 30169256

[46] Jones C, Ritchwood TD, Taggart T. Barriers and Facilitators to the Successful Transition of Adolescents Living with HIV from Pediatric to Adult Care in Low and Middle-Income Countries: A Systematic Review and Policy Analysis. AIDS Behav. 2019;23(9):2498–513. 10.1007/s10461-019-02621-6 31377893

[47] Sam-Agudu NA, Pharr JR, Bruno T, Cross CL, Cornelius LJ, Okonkwo P, et al Adolescent Coordinated Transition (ACT) to improve health outcomes among young people living with HIV in Nigeria: study protocol for a randomized controlled trial. Trials. 2017;18(1):595 10.1186/s13063-017-2347-z 29237487 PMC5729403

[48] Bangsberg DR. Less than 95% adherence to nonnucleoside reverse-transcriptase inhibitor therapy can lead to viral suppression. Clin Infect Dis. 2006;43(7):939–41. 10.1086/507526 16941380

[49] Murphy DA, Belzer M, Durako SJ, Sarr M, Wilson CM, Muenz LR. Longitudinal antiretroviral adherence among adolescents infected with human immunodeficiency virus. Arch Pediatr Adolesc Med. 2005;159(8):764–70. 10.1001/archpedi.159.8.764 16061785

[50] Merzel C, VanDevanter N, Irvine M. Adherence to antiretroviral therapy among older children and adolescents with HIV: a qualitative study of psychosocial contexts. AIDS Patient Care STDS. 2008;22(12):977–87. 10.1089/apc.2008.0048 19072104

[51] Weidle PJ, Wamai N, Solberg P, Liechty C, Sendagala S, Were W, et al Adherence to antiretroviral therapy in a home-based AIDS care programme in rural Uganda. Lancet. 2006;368(9547):1587–94. 10.1016/S0140-6736(06)69118-6 17084759

[52] Okatch H, Beiter K, Eby J, Chapman J, Marukutira T, Tshume O, et al Brief Report: Apparent Antiretroviral Overadherence by Pill Count is Associated With HIV Treatment Failure in Adolescents. J Acquir Immune Defic Syndr. 2016;72(5):542–5. 10.1097/QAI.0000000000000994 26990822 PMC4942380

[53] Dowshen N, D'Angelo L. Health care transition for youth living with HIV/AIDS. Pediatrics. 2011;128(4):762–71. 10.1542/peds.2011-0068 21930548

