# Supplementary figures and images for: Longitudinal evaluation of adherence, retention, and transition patterns of adolescents living with HIV in Nigeria
Source: PLoS One. 2020 Jul 31;15(7):e0236801. doi: 10.1371/journal.pone.0236801 (PMC7394430; doi:10.1371/journal.pone.0236801)

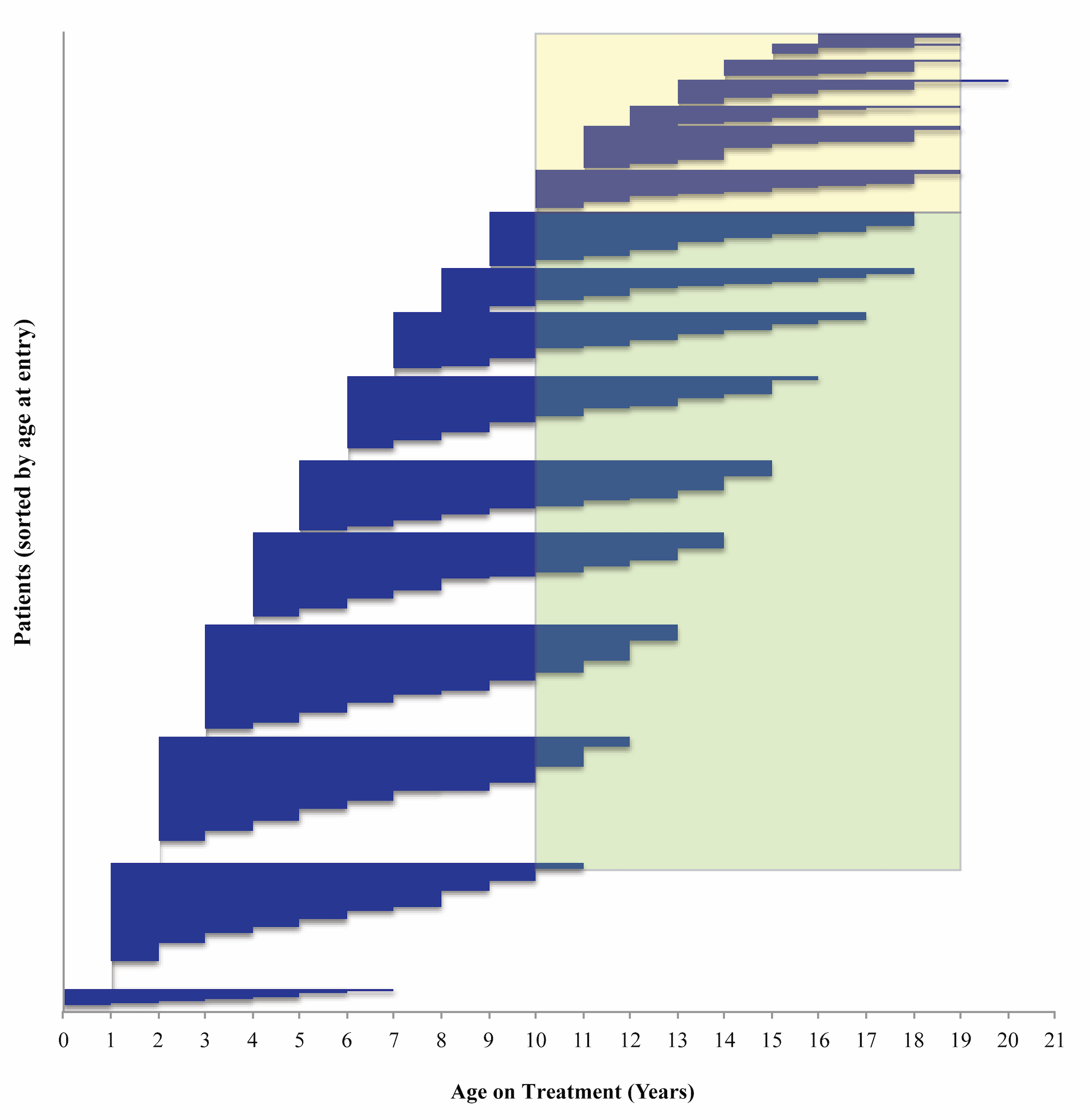

Supplement: S1 Fig — Shaded boxes highlight patients that received ART as adolescents; green shading highlights patients that initiated ART prior to adolescence and yellow shading highlights patients that initiated ART as adolescents. (TIF) [file pone.0236801.s001.tif]
